# NT Pro‐BNP can be used as a risk predictor of clinical atrial fibrillation with or without left atrial enlargement

**DOI:** 10.1002/clc.23760

**Published:** 2021-12-24

**Authors:** Xiao Zhao, Hao Li, Cai Liu, Yuanyuan Ren, Chaofeng Sun

**Affiliations:** ^1^ The Second Affiliated Hospital of Xi'an Jiaotong University Xi'an Shaanxi China; ^2^ Rehabilitation and Treatment Department The First Affiliated Hospital of Xi'an Jiaotong University Xi'an Shaanxi China; ^3^ Health Science Center of Xi'an Jiaotong University Xi'an Shaanxi China; ^4^ Medical Science Center of Yan'an University Yan'an Shaanxi China; ^5^ Cardiovascular Department The First Affiliated Hospital of Xi'an Jiaotong University Xi'an Shaanxi China

**Keywords:** atrial fibrillation, left atrial enlargement, NT Pro‐BNP, risk factor

## Abstract

**Background:**

NT Pro‐BNP is a blood marker secreted by cardiomyocytes. Myocardial stretch is the main factor to stimulate NT Pro‐BNP secretion in cardiomyocytes. NT Pro‐BNP is an important risk factor for cardiac dysfunction, stroke, and pulmonary embolism. So does atrial myocyte stretching occur when patients have atrial fibrillation (AF)? Whether atrial muscle stretch induced by AF leads to increased NT Pro‐BNP remains unclear. The purpose of this study is to investigate the relationship between NT Pro‐BNP and AF.

**Hypothesis:**

AF can cause changes in myocardial tension. Changes in myocardial tension may lead to increased secretion of NT Pro‐BNP. We hypothesize that NT Pro‐BNP may increase in AF with or without LAD enlargement.

**Methods:**

This clinical study is an observational study and has been approved by the Ethics Committee of the First Affiliated Hospital of Xi'an Jiaotong University. Ethical approval documents is attached. The study retrospectively reviewed 1345 patients with and without AF. After excluding 102 patients who were not eligible, the final total sample size was 1243 cases: AF group 679 patients (378, 55.7% males) and non‐AF group 564 patients (287, 50.8% males). NT Pro‐BNP was observed in AF group and non‐AF group with or without LAD. After adjusting for age, gender, BMI, left atrial diameter, hypertension, diabetes, coronary heart disease, and cerebral infarction, NT Pro‐BNP remains statistically significant with AF.

**Conclusion:**

NT Pro‐BNP can be used as a risk predictor of AF with or without left atrial enlargement.

## 1 INTRODUCTION

It is anticipated that over the next four decades the prevalence of atrial fibrillation (AF) will increase dramatically due to an aging population, improved therapies, and longer survival rate with heart disease.^1^, ^2^ AF is one of the most common arrhythmias in clinical practice^3^ and a major source of cardiovascular morbidity and mortality.^4^ AF is also associated with higher rates of stroke and hospitalization,^5^, ^6^ AF also reduces the quality of life, increases the risk of heart failure and worsens mortality,^7^ and is thought to account for nearly half of embolic strokes.^8^ Identifying risk factors of AF are important tasks for public health.^9^, ^10^ The supplement of risk factors related to AF are conducive to early identification, early intervention and treatment to prevent the occurrence of stroke. However, traditional risk factors do not predict the total risk of stroke in AF. Therefore, risk predictors need to be improved to further understand the pathophyphysiology of AF.^11^, ^12^ Blood biomarkers are potential tools for AF risk prediction and provide insights into the disease's pathophysiology. The 108 amino acid precursor molecule BNP is a polypeptide encoded by a gene on chromosome 1. Which is secreted from both the atria and the ventricles.^13^ The intracellular prohormone of brain natriuretic peptide (Pro‐BNP) is split into the biologically active brain natriuretic peptide (BNP) (the 32 amino‐acid of the C‐terminal fragment, biologically active BNP) and the remaining inactive N‐terminal fragment of Pro‐BNP (NT Pro‐BNP) (the 76 amino acid, biologically inactive NT Pro‐BNP). NT Pro‐BNP has a longer plasma half‐life than BNP and may provide better diagnostic value.^14^, ^15^ The main stimulus for cardiac NT Pro‐BNP secretion is myocardial stretch^16^, ^17^ and the pressure or volume overload.^18^ BNP is usually a marker of ventricular dysfunction. This hormone is released in response to ventricular stretch.^19^ Meanwhile, patients with pulmonary embolism were also observed to be associated with a significant increase in NT Pro‐BNP. NT Pro‐BNP is a significant risk factor for stroke of cardiac insufficiency and pulmonary embolism.^19^ Patients with normal levels of NT Pro‐BNP have low risks for death as well as for hemodynamic deterioration.^19^ AF is usually accompanied by changes in atrial stretch, which may also cause changes in NT Pro‐BNP secretion. Stroke and embolism are also risk factors for AF. The purpose of this experiment was to explore whether AF can cause changes in NT Pro‐BNP secretion and whether NT Pro‐BNP can be considered as a risk factor for the occurrence of AF.

## 2 METHOD

### 2.1 The experimental flow chart of AF and non‐AF patients

**Figure 1:**
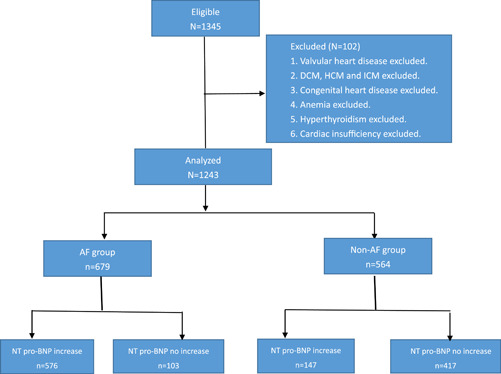
The above flow chart shows the design of inclusion and exclusion of AF and non‐AF groups. AF, atrial fibrillation

Figure 1 shows the entire process of the experiment.

### 2.2 The inclusion and exclusion criteria of AF and non‐AF patients

#### 2.2.1 Inclusion criteria for patients with AF

- More than two episodes of AF occurred and recorded by ECG before.
- Aged from 18 to 80 years old.

#### 2.2.2 Exclusion criteria for patients

Valvular Heart disease, cardiomyopathy, hyperthyroidism, anemia, congenital heart disease, and severe cardiac dysfunction (EF < 35%) were excluded.

### 2.3 The criterion of NT Pro‐BNP increase

The normal range of NT Pro‐BNP is 0‐125 pg/ml, once it is above 125 pg/ml, we define it as the increase of NT Pro‐BNP.

### 2.4 Case selection

This experiment aims to explore the role of NT Pro‐BNP in AF patients. A total of 1243 patients were enrolled from the Atrial Fibrillation Center, Department of Cardiology, the First Affiliated Hospital of Xi'an Jiaotong University. According to the inclusion and exclusion criteria, 679 patients with AF and 546 patients without AF were included. We also conducted subgroup analysis on the AF group and non‐AF group, we divided them into the NT Pro‐BNP increase group and the NT Pro‐BNP normal group separately according to the criterion of NT Pro‐BNP increased. When we are exploring the relationship between NT Pro‐BNP and AF, the influence of left ventricular diastolic diameter and left atrial diastolic diameter must be excluded. The relationship between NT Pro‐BNP and AF were observed when the diameter of the heart was within the normal range.

### 2.5 Statistical analysis

We used two independent sample *t*‐tests for continuous data and *χ* ^2^ tests for discontinuous data. Univariate analysis model is used for preliminary screening of related risk factors, and literature search is conducted to incorporate statistical factors into the multivariate logistic regression model for further analysis. Multivariate regression analysis is conducted on the premise of the establishment of the model and good fitting of the model.

## 3 RESULT

### 3.1 NT Pro‐BNP in AF and non‐AF groups

Through the Table 1, we observed that NT Pro‐BNP increased in the patients with AF compared with the non‐AF group.

**Table 1:** Univariate analysis of related factors in AF and non‐AF groups

Category	AF	Non‐AF	OR (95% CI)	*p* value
Gender (male)	Male (378, 55.7%) Female (301, 44.3%)	Male (287, 50.8%) Female (278, 49.2%)	1.212 (0.969–1.516)	*p* > .05
Age (*r*)	66.57 ± 11.92	57.90 ± 13.07	1.06 (1.05–1.07)	*p* < .001***
BMI (kg/m^2^)	25.08 ± 4.72	25.09 ± 3.32	1.00 (0.97–1.03)	*p* > .05
Heart rates (times/min)	77.64 ± 19.13	77.37 ± 12.79	1.00 (0.99–0.01)	*p* > .05
SBP (mm Hg)	125.59 ± 18.57	138.08 ± 24.49	0.97 (0.97–0.98)	*p* < .001***
DBP (mm Hg)	76.91 ± 14.58	83.01 ± 15.59	0.97 (0.97–0.98)	*p* < .001***
AST (U/L)	23.96 ± 14.22	23.55 ± 23.26	1.00 (1.00–1.00)	*p* >.05
ALT (U/L)	25.98 ± 25.25	26.92 ± 31.98	1.00 (1.00–1.00)	*p* > .05
CHOL (mmol/L)	4.35 ± 3.44	5.31 ± 4.36	0.93 (0.90–0.96)	*p* < .001***
TG (mmol/L)	1.32 ± 0.89	1.72 ± 2.54	0.71 (0.63–0.82)	*p* < .001***
LDL (mmol/L)	2.82 ± 19.66	2.33 ± 0.76	1.00 (0.99–1.01)	*p* > .05
HDL (mmol/L)	1.06 ± 0.37	1.32 ± 5.60	0.95 (0.72–1.26)	*p* > .05
BUN (mmol/L)	6.33 ± 2.87	5.60 ± 1.84	1.19 (1.11–1.27)	*p* < .001***
CRE (µmol/L)	69.04 ± 23.29	65.19 ± 26.61	1.01 (1.00–1.01)	*p* < .01**
CK (U/L)	86.76 ± 52.82	95.78 ± 98.42	1.00 (1.00–1.00)	*p* < .05*
CKMB (U/L)	13.30 ± 8.41	12.85 ± 15.08	1.00 (1.00–1.01)	*p* > .05
INR	1.43 ± 2.91	1.19 ± 3.92	1.03 (0.98–1.09)	*p* > .05
FDP (mg/L)	2.59 ± 9.35	1.44 ± 1.11	1.21 (1.10–1.32)	*p* < .01**
Hemoglobin a1c (%)	5.91 ± 0.83	5.73 ± 0.72	1.39 (1.18–1.63)	*p* < .001***
K^+^ (mmol/L)	4.61 ± 15.32	3.89 ± 0.41	2.23 (1.69–2.96)	*p* > .05
Na^+^ (mmol/L)	142.31 ± 39.42	142.79 ± 4.57	1.00 (1.00–1.00)	*p* > .05
Cl (mmol/L)	100.66 ± 9.96	100.26 ± 6.62	1.00 (0.99–1.01)	*p* >.05
FT4 (pmmol/L)	14.74 ± 3.70	14.72 ± 2.95	1.00 (0.97–1.04)	*p* > .05
FT3 (pmmol/L)	4.84 ± 6.56	4.79 ± 0.93	1.00 (0.98–1.03)	*p* > .05
TSH (uIU/L)	3.10 ± 4.82	2.78 ± 2.98	1.02 (0.99–1.06)	*p* > .05
QRS (s)	0.10 ± 0.02	0.10 ± 0.013	0.00 (0.00–0.03)	*p* < .01**
Left atrial diameter (mm)	38.83 ± 6.34	32.22 ± 3.89	1.32 (1.27–1.36)	*p* < .001***
Left ventricular diameter (mm)	46.21 ± 5.55	45.22 ± 4.50	1.04 (1.02–1.06)	*p* < .01**
EF (%)	64.52 ± 7.86	67.75 ± 5.61	0.93 (0.91–0.95)	*p* < .001***
CO (L/min)	5.94 ± 1.52	5.65 ± 1.23	1.16 (1.07–1.26)	*p* < .001***
NT Pro‐BNP (pg/ml)	Yes 626 (92.06%) No 54 (7.94%)	Yes 191 (33.93%) No 372 (66.07%)	22.43 (16.15–31.13)	*p* < .001***
Cerebral infarction	Yes 50 (7.4) No 628 (92.5)	Yes 8 (1.4) No 556 (98.6)	5.533 (2.601–11.773)	*p* < .001***
Hypertension	Yes 351 (51.7) No 328 (48.3)	Yes 455 (80.7) No 109 (19.3)	0.256 (0.198–0.332)	*p* < .001***
Diabetes	Yes 120 (17.7) No 559 (82.3)	Yes 80 (14.2) No 484 (85.8)	1.299 (0.955–1.767)	*p* > .05
Coronary heart disease (CHD)	Yes 145 (21.4) No 533 (78.5)	Yes 56 (9.9) No 508 (90.1)	2.463 (1.768–3.431)	*p* < .001***
Kidney disease (CKD)	Yes 9 (1.3) No 670 (98.7)	Yes 13 (2.3) No 551 (97.7)	0.569 (0.242–1.342)	*p* > .05

*Note*: Data shows single‐factor regression analysis of relevant factors.

### 3.2 Univariate analysis between AF group and non‐AF group

We collected the following factors: the patients' age, gender, weight, height, respiratory rate, pulse, heart rate, body temperature, blood pressure, blood routine examination, urine routine examination, liver function test, renal function test, thyroid function test, coagulation function test, electrocardiogram, cardiac ultrasound, Holter, drugs, and other factors. Univariate analysis was performed on factors that might be associated with AF. Univariate results are shown in Table 1: univariate analysis results show that the NT Pro‐BNP, age, systolic blood pressure, diastolic blood pressure, total cholesterol, triglycerides, urea nitrogen, creatinine, creatine kinase, glycosylated hemoglobin, fibrin degradation products, QRS interphase, left atrial diameter, left ventricular diameter, ejection fraction, cardiac output, cerebral infarction, hypertension, and coronary heart disease may be associated with AF (*p* < .05).

### 3.3 Multivariate logistic regression analysis between AF group and non‐AF group

We then selected related factors into the multivariate logistic regression model by consulting relevant literature and the results of univariate analysis. Finally, we selected NT Pro‐BNP, age, gender, BMI, hypertension, coronary heart disease, cerebral infarction, diabetes, and left atrial diameter into the multifactor logistic regression model.

Before the multifactor logistic regression analysis of the relevant factors, we first tested whether the multifactor regression model was reasonably established and whether the goodness of fit was suitable for the model. The results showed that after these factors were included, the model was established reasonably (the *p* value of omnibus test is *p* <.001) and the goodness of fit of the model was proper (the *p* value of HL test is *p* > .05).

As it is shown in Table 2: after we incorporate the above factors into the multifactor logistic regression model, the NT Pro‐BNP (95% confidence interval [CI]: 11.30 7.65–16.69, *p* < .001), left atrial diameter (95% CI: 1.22 1.18–1.27, *p* < .001), cerebral infarction (95% CI: 0.28 0.11–0.74, *p* < .05), hypertension (95% CI: 5.52 3.78–8.06, *p* < .001), coronary heart diseases (95% CI: 0.62 0.40–0.97, *p* < .05) are statistically significant. At the same time, after adjusting for age, gender, BMI, hypertension, coronary heart disease, cerebral infarction, diabetes, and left atrial diameter, the NT Pro‐BNP is still statistically associated with AF with a relative risk of 11.30 (95% CI: 11.30 7.65–16.69, *p* < .001).

**Table 2:** Multivariate regression of atrial fibrillation related factors

Category	Univariate analysis	Multivariate analysis regression	95% CI for Exp(*B*)
NT Pro‐BNP (pg/ml)	*p* < .001***	*p* < .001***	11.30 (7.65–16.69)
BMI	*p* > .05	*p* > .05	0.99 (0.95–1.03)
Age (*r*)	*p* < .001***	*p* > .05	1.01 (1.00–1.02)
Gender (male)	*p* > .05	*p* > .05	0.78 (0.55–1.11)
Left atrial diameter (mm)	*p* < .001***	*p* < .001***	1.22 (1.18–1.27)
Cerebral infarction	*p* < .001***	*p* < .05*	0.28 (0.11–0.74)
Hypertension	*p* < .001***	*p* < .001***	5.52 (3.78–8.06)
Coronary heart disease (CHD)	*p* < .001***	*p* < .05*	0.62 (0.40–0.97)
Diabetes	*p* >.05	*p* > .05	0.84 (0.54–1.29)

*Note*: Data shows the results of further statistical analysis by incorporating the factors with statistical significance into the multifactor analysis model.

### 3.4 Subgroup analysis with and without left ventricular enlargement

To rule out the influence of NT Pro‐BNP increase caused by ventricular enlargement. First, we defined whether the left ventricle was enlarged according to the normal range of left ventricular diameter at the end diastolic stage of cardiac ultrasound. Male patients with left ventricular end‐diastolic diameter greater than 55 mm and female patients with left ventricular end‐diastolic diameter greater than 50 mm were defined as the left ventricle enlargement. Second, we conducted subgroup analysis between the AF group and the non‐AF group, and divided them into four subgroups: the left ventricular enlargement group in AF, the left ventricular enlargement group in non‐AF, the left ventricular normal group in AF, the left ventricular normal group in non‐AF. The *χ* ^2^ test was performed to determine whether the left ventricle of the AF group and the non‐AF group increased (Table 3).

**Table 3:** Subgroup analysis of the NT Pro‐BNP with and without LVEDD enlargement

	AF	Non‐AF	*p* value
LVEDD (mm)	46.21 ± 5.55	45.22 ± 4.50	*p* > .05
LVEDD enlargerment	57	34	
LVEDD with no enlargerment	622	530	

Abbreviation: LVEDD, left ventricular end diastolic diameter.

According to the above Table 3, we found that the left ventricular end‐diastolic diameter of the AF group is 46.21 ± 5.55 mm. The left ventricular end‐diastolic diameter of the non‐AF group is 45.22 ± 4.50. In the AF group, 57 patients can be defined as having left ventricular end‐diastolic diameter enlargement. In the non‐AF group, 34 patients can be defined as having left ventricular end‐diastolic diameter enlargement. The *χ* ^2^ test results shows no statistically significance exist in left ventricular end‐diastolic diameter between the AF group and the non‐AF group (*p* > .05).

### 3.5 Subgroup analysis with and without left atrial enlargement

To further analyze whether the increase of NT Pro‐BNP in the AF group is caused by the enlargement of left atrium, or even if the left atrium did not enlarge, AF could still increase NT Pro‐BNP. Therefore, we conduct subgroup analysis between the AF group and the non‐AF group, we defined whether the left atrium was enlarged according to the normal range of left atrial diameter at the end diastolic stage of cardiac ultrasound. Patients with left atrial end‐diastolic diameter greater than 35 mm were defined as the left atrial enlargement. and then we divide them into four subgroups: the left atrial enlargement group in AF, the left atrial enlargement group in non‐AF, the left atrial normal group in AF, the left atrial normal group in non‐AF. Comparison was made between the left atrial enlargement group of AF patients and the left atrial enlargement group of non‐AF patients, between the left atrial normal group of AF patients and the left atrial normal group of non‐AF patients, and between the left atrial enlargement group of AF patients and the left atrial normal group of AF patients.

Subgroup analysis was performed on the AF group and non‐AF group as Table 4. We found a statistically significant increase in NT Pro‐BNP in the Left atrial enlargement group of AF compared with the Left atrial normal group in AF. Enlargement of the left atrium did lead to an increase in NT Pro‐BNP (*p* < .001).

**Table 4:** The analysis of left atrial enlargement subgroups in the AF and non‐AF groups

	NT Pro‐BNP increase	No NT Pro‐BNP increase	*p* value
Left atrial enlargement in AF	521	22	*p* < .001***
Left atrial enlargement in non‐AF	84	92	

	NT Pro‐BNP increase	No NT Pro‐BNP increase	
Left atrial normal in AF	103	31	*p* < .001***
Left atrial normal in non‐AF	8	380	

	NT Pro‐BNP increase	No NT Pro‐BNP increase	
Left atrial enlargement in AF	521	22	*p* < .001***
Left atrial normal in AF	103	31	

*Note*: Data shows an analysis of left atrial enlargement subgroups in the AF and non‐AF groups.

We compared the left atrial enlargement group in AF with the left atrial enlargement group in non‐AF, and found that the level of NT Pro‐BNP was significantly increased in the left atrial enlargement group in AF (*p* < .001). To avoid the interference of left atrial enlargement on the increase of NT Pro‐BNP, We compared the left atrial normal group in AF with the left atrial normal group in non‐AF, and the level of NT Pro‐BNP in the left atrial normal group in AF was also significantly increased (*p* < .001). In this subgroup, although AF did not cause left atrial enlargement, irregular, asynchronous atrial myocardial contraction during AF may still lead to changes in myocardial tone, which may also lead to increase of NT Pro‐BNP.

In conclusion, when AF was compared with the non‐AF group, AF was associated with an increase in NT Pro‐BNP regardless of the presence of left atrial enlargement.

## 4 DISCUSSION

AF is one of the most common arrhythmias in clinical practice^3^ and a major source for cardiovascular and cerebrovascular morbidity and mortality.^4^ AF is also associated with higher rates of stroke and hospitalization,^5^, ^6^ decreased quality of life,^7^ increased risk of heart failure and with the increase of mortality. Moreover, AF is considered to account for nearly half of all embolic strokes.^8^ Identifying risk factors for AF is an important task for public health.^9^, ^10^ The supplement the risk factors of AF is conducive to early identification, early intervention and early treatment for AF to prevent the occurrence of stroke and embolism. Biomarkers in the blood are potential tools for predicting AF risk and providing insights into the pathophysiology of the disease. NT Pro‐BNP may provide a better diagnostic resolution.^14^, ^15^

In the past, BNP has emerged as a powerful diagnostic tool for detecting acute heart failure and left ventricle systolic and/or diastolic dysfunction.^20^, ^21^ High BNP level is associated with left atrial auricle thrombosis,^22^ and may also be a predictor of thromboembolism in patients with pulmonary embolism. Patients with normal NT Pro‐BNP levels have a lower risk of death and hemodynamic deterioration leading to any adverse events.^19^ However, with the development of research in recent years, it has been reported that the NT Pro‐BNP is also produced in the atrial wall,^17^ the main stimulus for cardiac NT Pro‐BNP secretion is myocardial stretch;^16^ AF is usually associated with changes in atrial muscle tone and may also lead to changes in NT Pro‐BNP secretion. Therefore, we hypothesized whether NT Pro‐BNP could be used as a risk predictor of AF. The purpose of this study is to investigate whether AF can cause the increase of NT Pro‐BNP and whether NT Pro‐BNP can be considered as a risk factor for the occurrence of AF. This study systematically reviewed 1243 patients with and without AF. We found that NT Pro‐BNP was increased in the AF group compared with the non‐AF group (*p* < .001).

To exclude the interference of increased NT Pro‐BNP caused by left ventricular enlargement, we first needed to verify whether there were differences in left ventricular enlargement between the AF and non‐AF groups. Therefore, the end diastolic diameter of the left ventricle in the AF group and the non‐AF group was statistically analyzed, and there was no statistical difference between the two groups (*p* > .05).

After excluding the interference of left ventricular enlargement on NT Pro‐BNP, we needed to further investigate the effect of left atrial enlargement on NT Pro‐BNP secretion in patients with AF. Whether the increase of NT Pro‐BNP in AF group was caused by left atrial enlargement, or whether AF could still increase NT Pro‐BNP even without left atrial enlargement.

Therefore, Subgroup analysis was performed on the AF group and non‐AF group. We found that compared with the Left atrial normal group in AF, the Left atrial enlargement group in AF experienced an statistical increase in NT Pro‐BNP (*p* < .001). Enlargement of the left atrium leads to an increase in NT Pro‐BNP.

The main stimulus for cardiac NT Pro‐BNP secretion is myocardial stretch. The enlargement of the left atrium usually stimulates the increase of NT Pro‐BNP. The experimental results also prove that when the left atrium expands, NT Pro‐BNP increases. However, how does the level of NT Pro‐BNP change in AF patients without left atrial enlargement? To further investigate this problem, we compared the left atrial normal group in AF with the left atrial normal group in non‐AF, and found that the level of NT Pro‐BNP in the left atrial normal group in AF was also statistically higher than that in the non‐AF group (*p* < .001).

Through the above research results we found that even without obvious left atrial enlargement of AF, minor, irregular and asynchronous atrial myocardial stretch during AF may still lead to changes in myocardial stretch, which may also lead to the increase of NT Pro‐BNP.

The results of this experiment showed that: regardless of the presence or absence of enlarged left atrium, the NT Pro‐BNP in the AF group was significantly higher than that of the non‐AF group. NT Pro‐BNP can be a risk factor for the occurrence of AF, no matter with or without the influence of enlarged left atrium.

## 5 CONCLUSION

NT Pro‐BNP can be used as a risk predictor of AF with or without left atrial enlargement.

## 6 LIMITATION

As an observational study, this study has limitations, including the data is obtained from a single center, the data is not representative enough, the sample size of the data needs to be expanded, and some data are missing. However, the whole 1243 cases were all obtained from the our hospital after ethical approval, which could represent our team's overall interests.

## CONFLICT OF INTERESTS

The authors declare that there are no conflict of interests.

## Data Availability

Patients' data are protected by hospital ethics committees, and the data that support the findings of this study are available from the unanimous consent of hospital ethics committees and corresponding author upon reasonable request.
